# Ruthenium and Platinum-Modified Titanium Dioxide Support for NaBH_4_ Hydrolysis

**DOI:** 10.1021/acsomega.3c04269

**Published:** 2023-09-20

**Authors:** Cigdem Tuc Altaf, Valentina G. Minkina, Stanislav I. Shabunya, Tuluhan O. Colak, Nurdan Demirci Sankir, Mehmet Sankir, Vladimir I. Kalinin

**Affiliations:** † Micro and Nanotechnology Graduate Program, TOBB University of Economics and Technology, Sogutozu Caddesi no. 43, Sogutozu 06560, Ankara, Turkey; ‡ A.V. Luikov Heat and Mass Transfer Institute of the National Academy of Sciences of Belarus, P. Brovka, 15. Minsk 220072, Republic of Belarus; § Department of Materials Science and Nanotechnology Engineering, TOBB University of Economics and Technology, Sogutozu Caddesi no. 43, Sogutozu 06560, Ankara, Turkey

## Abstract

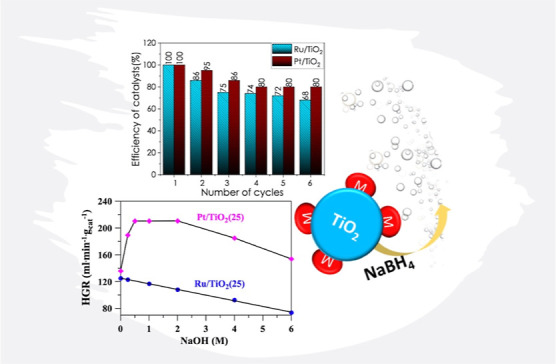

Highly stable platinum (Pt) and ruthenium (Ru)-based catalysts on titanium oxide (TiO_2_) nanoparticle support were prepared. The productivity of hydrogen generation from sodium borohydride (NaBH_4_) hydrolysis was observed to be as high as 95%. The activation energies for the hydrolysis reaction in the presence of Ru/TiO_2_ in aqueous and alkaline solutions were 62.00 and 64.65 kJ mol^–1^, respectively. On the other hand, the activation energy value of the hydrolysis reaction with the Pt/TiO_2_ catalyst decreased from 60.5 to 53.2 kJ mol^–1^, and the solution was changed from an aqueous to an alkaline medium. The experimental results have indicated that NaOH concentration (ranging from 0.5 to 2 M) affected the hydrogen generation rate (HGR) differently for both metals on the TiO_2_ support. Consequently, the HGR of the hydrolysis reaction in the presence of the Ru/TiO_2_ catalyst decreased with increasing NaOH concentration, whereas the Pt/TiO_2_ catalyst efficiency increased with increasing NaOH concentration.

## 1 Introduction

Chemical hydrides are regarded as promising materials for storing and supplying hydrogen gas to fuel cells. Particularly, sodium borohydride (NaBH_4_) stands out as a good candidate among all with its high hydrogen storage capacity (up to 10.8 wt % in gravimetric density).^1–4^ Hydrogen production via the NaBH_4_ hydrolysis process offers several advantages, such as high-purity hydrogen forming at low temperatures with the environmentally safe byproduct sodium metaborate (NaBO_2_). In addition, the use of a catalyst allows for the acceleration of the production of hydrogen.^5–7^ All these characteristics make NaBH_4_ an attractive hydrogen storage material for portable applications.^8–11^ Numerous catalysts, including non-noble and noble metals, metal oxides, and carbon-based nanomaterials, have been used so far to achieve efficient NaBH_4_ hydrolysis.^5,12–14^ Although non-noble metal catalysts are cost-effective, they are still inferior to noble metal catalysts in terms of performance. Thus, the utilization of support catalysts has been studied in the literature to relegate the use of noble metals without forfeiting their efficiency.^15–17^

As Supporting Information, TiO_2_ prevents the agglomeration of active particles and provides a surface to build a heterogeneous catalyst with higher activity in various catalytic systems.^18,19^ In the past decade, titanium dioxide (TiO_2_) has gained attention for its role in the catalytic hydrolysis of NaBH_4_.^20–25^ So far, various metals and metal oxide materials, such as cobalt borates, cobalt, nickel, samarium, and cerium oxide, have been used with TiO_2_ support for hydrogen production from NaBH_4_ hydrolysis.^20–24,26,27^ On the other hand, a relatively limited number of studies have been performed on the use of Ru/TiO_2_ and especially Pt/TiO_2_ catalysts in NaBH_4_ hydrolysis.^15,28–31^ In addition, a comparative analysis is lacking for the activation energies of the catalysts based on Ru and Pt on TiO_2_ support in aqueous and alkaline solutions.^32–34^ Most of the literature reports on activation energy comprise carbon-based support materials for Ru and Pt metals.^32–34^ In previous research based on TiO_2_-supported Ru nanoparticles, Wei et al. reported an H_2_ generation in the NaBH_4_ hydrolysis reaction with an activation energy of 55.9 kJ mol^–1^.^31^ In recent work, the synergetic effect of porous titanium oxide cages was highlighted for PtNi alloy nanoparticles to have very low activation energy (28.7 kJ mol^–1^).^35^

Moreover, there is still a conflict regarding the effect of the NaOH addition in the literature. Most authors point out that HGR decreases with increasing NaOH concentration in solution for both platinum^36–39^ and ruthenium^32,37–43^ catalysts, regardless of the nature of the carrier. During our literature search, we have encountered only a few articles in which HGR increases with rising NaOH concentrations when Ru-based catalysts are used. In two independent studies by Walter et al.,^44^ using elemental Ru and Tuan and Lin^45^ using Ru/ZIP catalysts, the HGR values reached the highest rate with increasing NaOH concentration. The authors explained their observations with Ru/ZIF, containing both Ru and Co catalysts, which had a very high surface area and displayed stability in water at high temperatures.^45^ This explanation is logical since all available articles report that HGR increases with increasing NaOH concentration in the presence of Co-based catalysts on any carriers.^23,46–51^ On the other hand, only low concentrations of NaOH (0–0.11 M) addition were investigated for Co–Ru/C catalysts by Huang et al., suggesting that the best efficiency was obtained in the presence of 0.09 M of NaOH with a drop in activation energy from 60.09 to 50.20 kJ mol^–1^.^52^ Bozkurt et al. conducted two experiments with a Pt/Co_3_O_4_ catalyst in 1 and 10 wt % NaOH solutions, and that indicated a decrease in HGR with increasing NaOH concentration.^53^ In research conducted by Kang et al., a comparison of the catalytic activities of some metal nanoparticles, including Ru, Pt, Ni, Co, and Cu, exposed that the low concentration of NaOH (0.1–0.4 M) addition had a positive effect on all metal nanoparticles except Pt.^54^ Thus, in the present study, the efficiency of prepared catalysts based on Pt and Ru-modified TiO_2_ carriers was compared since the rate of hydrogen production mainly depends on the activity of the catalysts. Except for the type of metal catalyst on the support material, other factors, such as the temperature of the reaction solution and the concentration of NaBH_4_ and NaOH, could play significant roles in NaBH_4_ hydrolysis. These factors have been studied and discussed in this work.

## 2 Experimental Section

### 2.1 Materials

The chemicals used in this work were of analytical grade and used without further purification. The syntheses were carried out in Millipore Milli-Q ultrapure water as a medium. Chloroplatinic acid hydrate (H_2_PtCl_6_·H_2_O, Sigma-Aldrich, 99.9% trace metal) and ruthenium trichloride (RuCl_3_·3H_2_O, Sigma-Aldrich, technical) were used as metal nanoparticle sources. Commercial titanium (IV) oxide (TiO_2_, P-25 Degussa, Sigma-Aldrich, 21 nm, 99.5% trace metal basis) was used as the support material. Sodium borohydride (NaBH_4_) was used as a reducing agent and for kinetic experiments.

### 2.2 Synthesis of the Catalysts

Ru/TiO_2_ and Pt/TiO_2_ catalysts were synthesized by impregnation chemical reduction of the corresponding metal salt on the surface of TiO_2_ using NaBH_4_ as a reducing agent. Briefly, the desired amount of Ru metal salt (0.13 g of RuCl_3_·3H_2_O) was dissolved in 100 mL of distilled water. 1.5 g of TiO_2_ and 2.5 g of citric acid were added and stirred for 1 h. A 10 mL, 1.32 M cooled aqueous solution of (0.5 g, 13.2 mmol) NaBH_4_ was added drop by drop to reduce Ru ions. We refrigerated distilled water for several minutes before dissolving NaBH_4_ because it reacts violently and rapidly with water at room temperature. As a result, the metal ion reduction on the TiO_2_ support surface was slowed down sufficiently by the cold NaBH_4_ solution to prevent any impurities from forming. After 1 h of stirring, the reduced residue was collected by centrifuging at 9000 rpm for 30 min, washed with distilled water 3 times, and dried at 60 °C in a vacuum oven for further use. The synthesis of Pt/TiO_2_ was conducted using citric acid as a stabilizing agent. 0.075 g of H_2_PtCl_6_·6H_2_O was dissolved in 100 mL of distilled water at room temperature. 5 mg of citric acid and 1.5 g of TiO_2_ powders were added and stirred for 1 h, followed by ultrasonication for 10 min. For the reduction process, a cooled solution of 0.034 g of NaBH_4_ (0.89 mmol, 1 mL) was added dropwise and stirred for an additional 1 h. The resulting product was centrifuged at 9000 rpm for 30 min and dried at 60 °C in a vacuum for further use. The experimental procedure is summarized in the flowchart in Figure S1.

### 2.3 Kinetic Experiments

Since the hydrogen produced by hydrolyzing NaBH_4_ is quite fluid, the kinetic studies had to be conducted in a very tightly sealed reactor. All units and parts were made of stainless steel. The volume of the reactor was 182.5 cm^3^, and the diameter was 5 cm. A specified temperature of *T*_0_ was set in the thermostat and a reactor with dry NaBH_4_ powder and a catalyst was placed in it. After heating the reactor to the temperature of *T*_0_, distilled water/alkaline solution was injected with a syringe, and the reactor was sealed. The temperature of the solution inside the reactor was measured with a Pt100 platinum resistance sensor (Autonics, Korea). Electronic sensors for operating pressures of 2.5, 10, 25, and 50 bar (Keller, Switzerland) were used as pressure gauges. The temperature of the solution and the pressure in the reactor were measured from the point in time when a water/alkaline solution was added to the end of hydrolysis. In all experiments, 50 mg of catalyst and 10 mL of distilled water or alkaline solution were used. The experiments were carried out in the temperature range of 20–60 °C, with NaBH_4_ molar concentrations of 0.265–4.23 M NaBH_4_ and alkali concentrations of 0–6 M NaOH. Two functions were measured in the experiments—the pressure in the reactor, which was proportional to the amount of released hydrogen, and the solution temperature. Using these measurements, the amount of hydrogen formed, the degree of decomposition ξ(*t*), and the rate of hydrogen formation were calculated.^19,55,56^

## 3 Results and Discussion

### 3.1 Catalyst Characterizations

**Figure 1:**
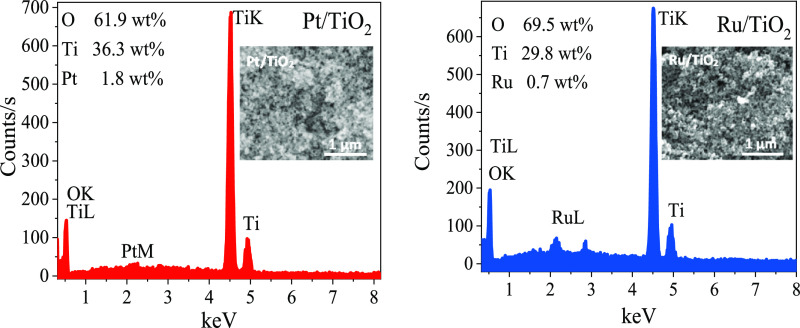
EDS spectra and the elemental weight ratio of the catalyst powder (inset is the SEM image).

Scanning electron microscopy (QUANTA 400 F Field Emission SEM) and energy-dispersive spectroscopy (EDS) analyses were performed to identify the microstructure elemental compositions in the catalysts (Figure 1). One of the remarkable differences between the elemental compositions of the two catalysts is the percentage of oxygen present in each catalyst. A higher oxygen content in Ru/TiO_2_ compared to Pt/TiO_2_ was observed. This can be explained by the formation of oxide compounds of ruthenium during reduction reactions.^57,58^ In addition, 1.8 and 0.7 wt % metallic Pt and Ru, respectively, were detected on the TiO_2_ structure in EDS analysis. The fact that these weight percentages are low is important in terms of reducing the use of expensive noble metals. While the oxygen contents are different, as seen in the insets of Figure 1, the surface morphologies of the samples are similar.

BET analysis of the pristine TiO_2_ support and catalyst powders before and after first use in NaBH_4_ is given in Supporting Information, Figures S2 and S3. The BET analysis results of pristine TiO_2_ powder given in Figure S2 confirm that the specific surface area of pristine TiO_2_ powders was calculated at 50 m^2^ g^–1^, as reported in the literature.^59,60^Figure S3a,f displays the BET-specific surface area of the prepared catalysts. The BET-specific surface areas for both catalysts were almost identical at 63 and 60 m^2^ g^–1^ for Pt/TiO_2_ and Ru/TiO_2_, respectively. This situation is compatible with the small amount of Pt and Ru incorporation into the TiO_2_ structure, as seen in the EDS analysis. Therefore, with the addition of Ru and Pt metal catalysts, there was an increase of approximately 20% in the specific surface area of the TiO_2_ powders. However, a drop in specific surface area after the first cycle of NaBH_4_ hydrolysis was observed for all samples and was 45 and 34 m^2^ g^–1^ for Pt/TiO_2_ and Ru/TiO_2_, respectively (Figure S3g–l).

### 3.2 NaBH_4_ Hydrolysis Experiments

**Figure 2:**
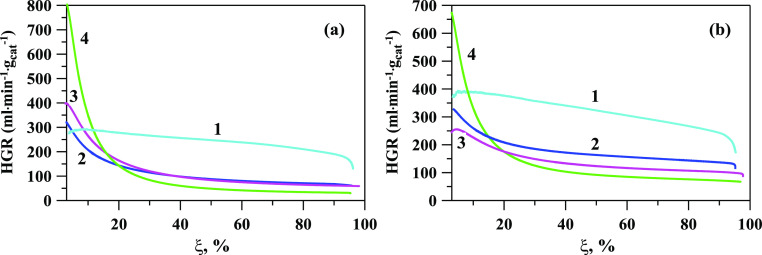
Effect of NaBH_4_ concentration on the HGR at 30 °C with (a) Ru/TiO_2_ and (b) Pt/TiO_2_. 1–0.265 M, 2–1.06 M, 3–2.126 M, and 4–4.23 M NaBH_4_.

The effect of the NaBH_4_ concentration on the hydrogen generation rate was investigated at 30 °C using Ru/TiO_2_ and Pt/TiO_2_ catalysts (Figure 2). The HGR relative to the degree of breakdown of NaBH_4_ is a more intuitive variable to study than the volume of hydrogen created as a function of time ξ(*t*). This representation is convenient for analyzing experiments of different durations because the function ξ(*t*) changes monotonically from 0 to 100%. Increases in catalyst concentration led to greater rates of hydrogen generation per unit of time or more “power” of heat release. Since heat production is proportional to catalyst load, only a small amount of catalyst should be utilized in kinetic studies if the heterogeneous hydrogen generation is much larger than the homogeneous hydrogen generation. Therefore, 50 mg of catalyst was used in these reactions.

As can be seen in Figure 2, the hydrogen generation rate decreases with an increase in the concentration of NaBH_4_ from 0.265 to 4.23 M for both catalysts. With increasing concentrations of NaBH_4_ solution, the concentration of sodium metaborate (NaBO_2_) as a byproduct rises, which leads to an increase in solution viscosity. The measurements of the viscosity of solutions have been conducted using a viscometer VIR-78 with an accuracy of ±3%. At 20 °C, the viscosity of 1.06 M of NaBH_4_ solution is about 1.25 MPa·s, after complete hydrolysis, the viscosity of the solution is 1.98 MPa·s. At 20 °C, the viscosity of 4.23 M NaBH_4_ solution is about 1.36 MPa·s; after complete hydrolysis, the viscosity of the solution is 5.77 MPa·s. The effect of solution viscosity on the HGR during hydrolysis of NaBH_4_ was previously reported in the literature and is confirmed by values of solution viscosity before and after the hydrolysis process.^43,61^ In addition, at a high concentration of NaBH_4_, the resulting NaBO_2_ may block the active centers of the catalysts. Thus, a higher concentration of NaBH_4_ makes it possible to achieve a higher hydrogen capacity, but it is limited by the solubility of NaBH_4_ and the hydrolysis product NaBO_2_ in water.

**Figure 3:**
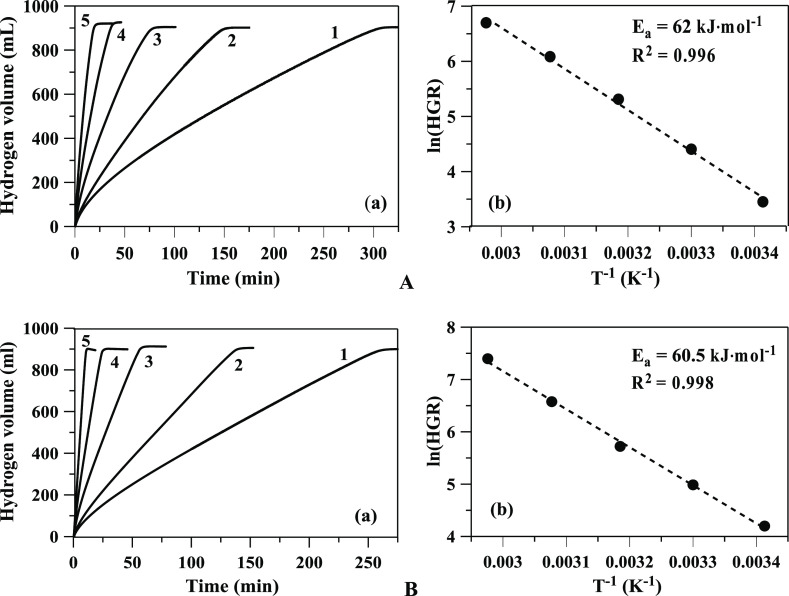
(a) Time evolution of hydrogen generation and (b) Arrhenius graph of NaBH_4_ catalytic hydrolysis in aqueous solutions with (A) Ru/TiO_2_ and (B) Pt/TiO_2_ [1–20, 2–30, 3–40, 4–50, and 5–60 °C (50 mg of catalyst)].

The kinetics of the hydrolysis reaction were studied within the temperature range of 20–60 °C in aqueous solutions with a molal concentration of 1.06 M NaBH_4_ (Figure 3). When determining the activation energy, we used linear approximations of the kinetic curves within the range of 10 to 85% of NaBH_4_ decomposition. The calculated effective rate constants of the zero-order reaction were used to obtain an approximation of the Arrhenius coefficients. The activation energies for Ru/TiO_2_ and Pt/TiO_2_ catalysts were 62.0 and 60.5 kJ mol^–1^, respectively (Figure 3b). These results are in agreement with the values obtained from two individual research works^32,33^ using the 10 wt % (Pt-Ru)@PVP catalyst (63.2 kJ mol^–1^) and using the 3 wt % Ru/C catalyst (64.5 kJ mol^–1^).

**Figure 4:**
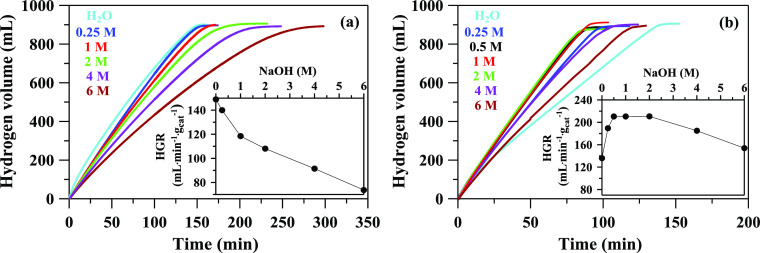
Effect of NaOH concentration on the time of complete hydrolysis at 30 °C with (a) Ru/TiO_2_ and (b) Pt/TiO_2_. Inset: HGR as a function of NaOH concentration (1.06 M NaBH_4_, 50 mg catalyst).

The results of our studies on the effect of adding alkali to the NaBH_4_ solution are shown in Figure 4. The NaOH concentration affects the HGR with Ru/TiO_2_ and Pt/TiO_2_ catalysts differently. The HGR for the Ru/TiO_2_ catalyst decreases with increasing NaOH concentration, while the HGR for the Pt/TiO_2_ catalyst increases in the range of 0.5–2 M NaOH, where the values are almost constant.

As a result, we may conclude that the influence of alkali on the rate of hydrolysis with Ru- and Pt-based catalysts is quite different. Only neutral particles involved in hydrolysis are adsorbed on the surface of the catalyst. In the NaBH_4_ solution, these can be water molecules and a complex [BH_4_^–^H^+^]. In the case of the Pt/TiO_2_ catalyst, it was assumed that hydrogen generation occurs on water molecules adsorbed by the catalyst. Within the framework of this hypothesis, water molecules in an adsorbed state become active; therefore, irreversible reactions of hydrogen generation are accelerated (Figure 4b). An increase in the concentration to 1 M NaOH leads to a slight increase in the total number of particles in the solution. At the same time, in an alkaline solution, the reduction of the proton concentration causes a significant decrease in the concentration of the complex. In this case, mainly H_2_O molecules are adsorbed on the surface of the catalyst, and as a result, the productivity of the generated hydrogen increases. An increase in the concentration to 4 M NaOH or more leads to a noticeable increase in the total number of particles in the solution. The collision of particles with the surface of the catalyst leads to the desorption of H_2_O molecules, and the larger the number of particles, the higher the probability of desorption. In addition, an increase in the concentration of alkali leads to an increase in hydrated complexes and the viscosity of the solution, but convection slows down. Therefore, this leads to a decrease in HGR. In the case of the Ru/TiO_2_ catalyst, it can be assumed that hydrogen generation occurs on the complex adsorbed by the catalyst. It can be assumed that OH^–^ ions remove complexes adsorbed on the surface of the catalyst. Since the number of complexes on the surface of the catalyst decreases, the addition of NaOH will lead to a decrease in HGR (Figure 4a).

**Figure 5:**
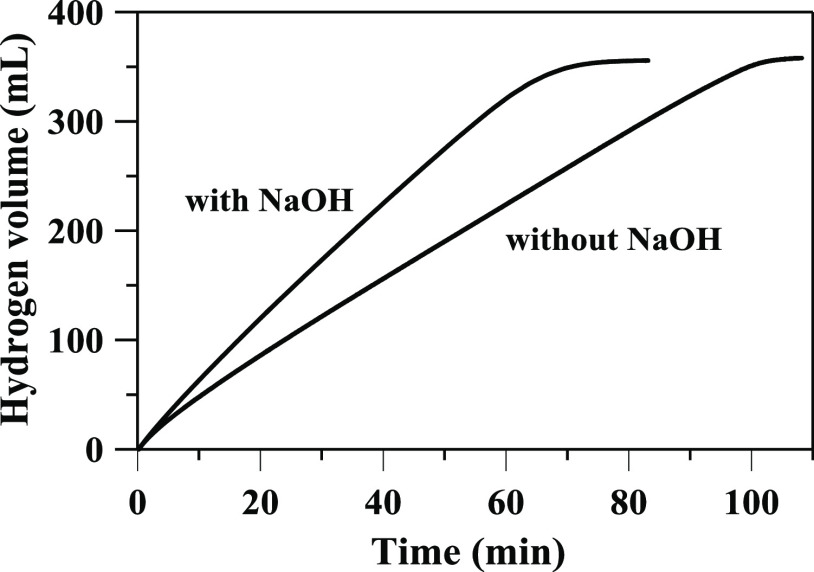
Effect of NaOH on the hydrogen generation at 20 °C with 1.06 M NaBH_4_, 1 M NaOH, and 50 mg of Pt/TiO_2_ catalyst.

Demirci et al. compared the rate of hydrolysis in aqueous and aqueous-alkaline solutions of NaBH_4_ with a Pt/ZS catalyst and stated that alkali addition negatively affects HGR.^39^ Experiments with our Pt/TiO_2_ catalyst under conditions similar to the previous work^39^ (0.42 M NaBH_4_, 20 °C, without/with 1 M NaOH) showed that the addition of NaOH leads to an acceleration of the hydrolysis process, and the HGR without/with NaOH is 72 and 107 mL min^–1^ g_cat_^–1^ respectively (Figure 5). Thus, we can assume that the nature of the influence of alkali on the rate of hydrolysis with Pt-based catalysts is determined by the carrier material.

**Figure 6:**
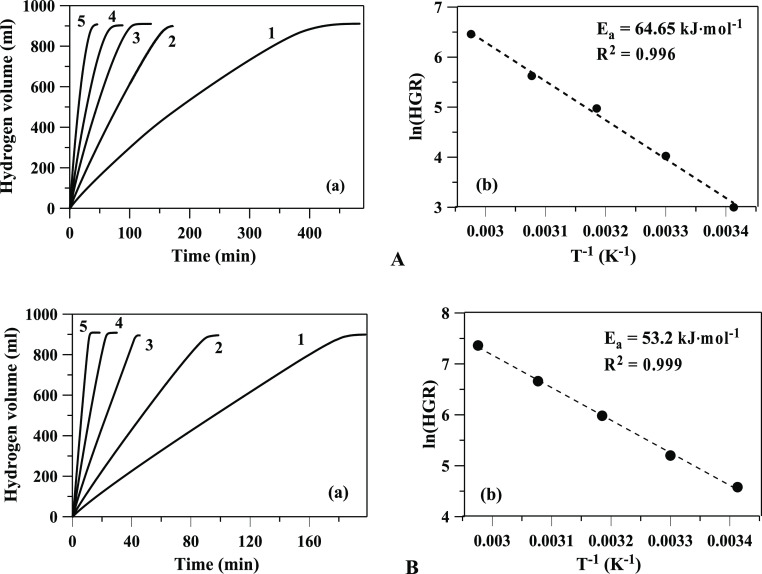
(a) Time evolution of hydrogen generation and (b) Arrhenius graph of NaBH_4_ catalytic hydrolysis in solutions with 1 M NaOH with (A) Ru/TiO_2_ and (B) Pt/TiO_2_ [1–20, 2–30, 3–40, 4–50, and 5–60 °C (1.06 M NaBH_4_, 50 mg of the catalyst)].

Figure 6 shows the hydrogen generation times from NaBH_4_ hydrolysis using Ru/TiO_2_ and Pt/TiO_2_ catalysts with 1 M NaOH addition that vary with temperature in the range of 20–60 °C. The calculated activation energies in the presence of Ru/TiO_2_ and Pt/TiO_2_ catalysts were 64.65 and 53.2 kJ mol^–1^, respectively. The pressure and temperature histories for both catalysts during the hydrolysis process are given in Figure S4. Since heat generation is proportional to catalyst load, we used a minimum amount of catalyst in kinetic studies, provided that the heterogeneous generation of hydrogen significantly exceeds the homogeneous one. Small deviations of temperature from linearity are observed in the experiments at 50 and 60 °C. When determining the activation energy, a correction was made for the temperature, which was 1–3° higher than in the thermostat

**Figure 7:**
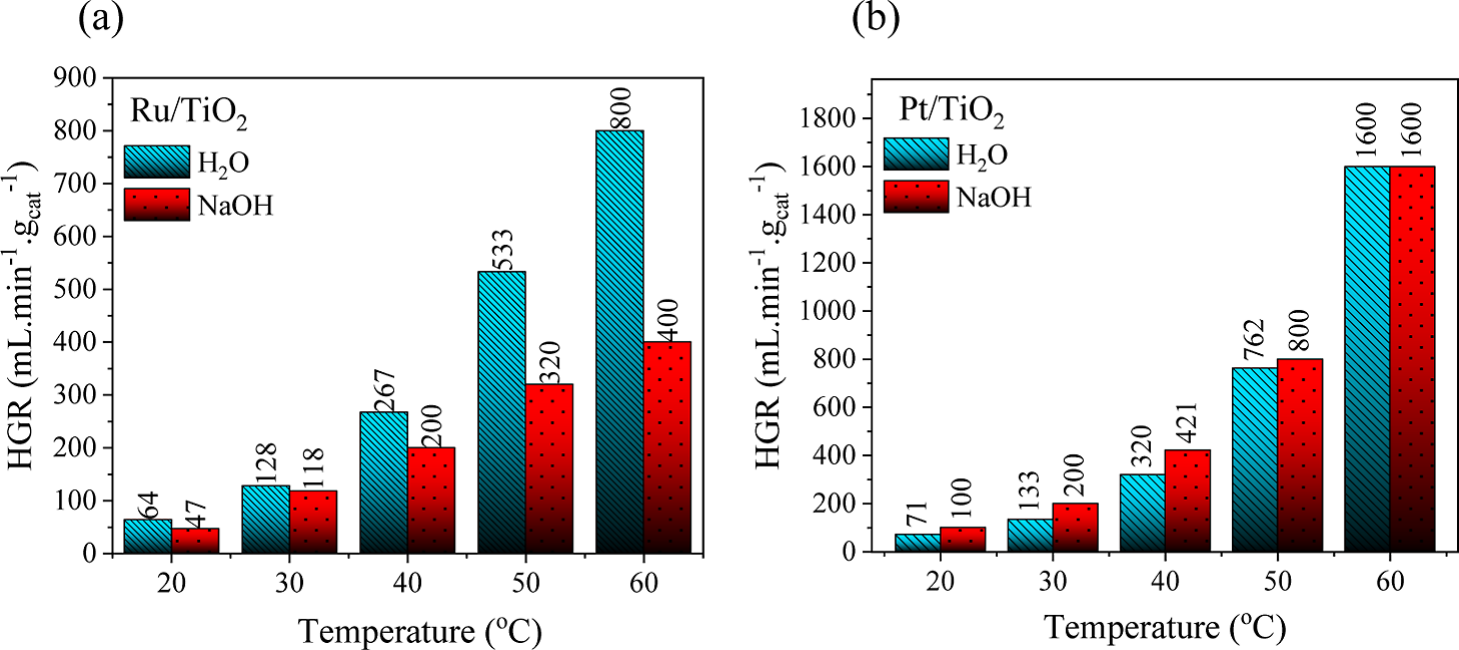
Effect of temperature on the average HGR in aqueous and alkaline solutions with (a) Ru/TiO_2_ and (b) Pt/TiO_2_ catalysts.

Figure 7 shows the effect of temperature in the range of 20–60 °C on the HGR using Ru/TiO_2_ and Pt/TiO_2_ catalysts in aqueous and alkaline NaBH_4_ solutions. A comparison of the hydrolysis process in aqueous and alkaline solutions with the Pt/TiO_2_ catalyst shows that the alkaline effect weakens with increasing temperature, whereas with the Ru/TiO_2_ catalyst, the alkaline effect on HGR increases. Following the EDS analysis results given in the inset of Figure 1, the incorporation of Pt metal is almost twice as high as that of Ru, supporting outperforming NaBH_4_ hydrolysis in the presence of Pt/TiO_2_.

The lower value of the activation energy for the Pt/TiO_2_ catalyst is evidence of its increased efficiency when alkali is added. At the same time, the calculated activation energy is not an absolute characteristic of the catalyst, which follows from the HGR data presented in Table S1. Commonly accepted techniques applied for kinetic data processing to determine the activation energy assume the functional dependence of HGR on temperature only. But activation energy latently depends on the sorption properties of the surface and the solution concentration.^55^ This is confirmed by the results presented in articles by Amendola et al.^40,62^ from which the dependence of the activation energy on the concentration of the solution follows. So, with 5 wt % Ru/IRA-400 catalyst for 7.5 wt % NaBH_4_–1 wt % NaOH solution, the activation energy is 56 kJ mol^–1^, and for 20 wt % NaBH_4_–10 wt % NaOH solution, it is −47.6 kJ mol^–1^. As expected, the dependence of the activation energy on the solution concentration manifested itself. In the article by Uzundurukan and Devrim^34^ for an aqueous solution of 3.82 wt % NaBH_4_ with 20 wt % Pt/C catalyst, the activation energy and the HGR are 36 kJ mol^–1^ and 4150 mL min^–1^ g_cat_^–1^, respectively; with a 20 wt % Pt/MWCNT catalyst −27 kJ mol^–1^ and 940 mL min^–1^ g_cat_^–1^, respectively. Thus, the dependence of the activation energy on the sorption properties of the surface manifested itself.

Although the Ru/LiCoO_2_ and Pt/LiCoO_2_ catalysts have shown high efficiency^37^ (see Table 1), at the same time, cyclic stability tests show that the activity of the catalysts decreases dramatically, and starting from the fourth cycle, the rate of hydrogen formation is comparable to the initial rate of LiCoO_2_.^63^ But one of the important tasks of researchers is to develop catalysts with good durability.

**Table 1:** Comparison Table of Activation Energies and HGRs During Hydrolysis of NaBH4 Catalyzed by Pt and Ru Catalysts

catalyst	Me (Ru, Pt) (wt %)	NaBH_4_ (wt %)	NaOH (wt %)	Cat. (mg)	*E*_a_ kJ·mol^–^^1^	average HGR at 25°C (mL min^–^^1^ g_cat_^–1^)	refs
Ru/C	3	3.18 M	0	300	61.2	318	(32)
Pt/MWCNT	20	3.82	0	20	27	940	(34)
Pt/C	20	3.82	0	20	36	4150	(34)
Ru/LiCoO_2_	1	10	5	20	68.5	3000	(37)
Pt/LiCoO_2_	1	10	5	20	70.4	2700	(37)
Ru/Co_3_O_4_	20	10	1	50	28.26	6514	(38)
Pt/Co_3_O_4_	20	10	1	50	43.52	4713	(38)
Ru/IRA-400	5	7.5	1	250	56	19	(40)
Ru/IRA-400	5	20	10	250	47.6	200	(62)
Ru/G	3	5	5	100	61.1	333	(66)
Ru/C	3	1	3.75	200	66.9	190	(67)
Ru/C	2	5	2	200	50.74	285	(43)
Ru/IR-120	1	5	1	200	49.72	132	(41)
Ru_2_Pt_1_/TiO_2_	1	0.52 M	1 M	250	NA	150 (20°C)	(68)
Pt/TiO_2_(25P)	1.79	1.06 M	1 M	50	53.2	140	this work
Ru/TiO_2_(25P)	0.62	1.06 M	0	50	62	100	this work

**Figure 8:**
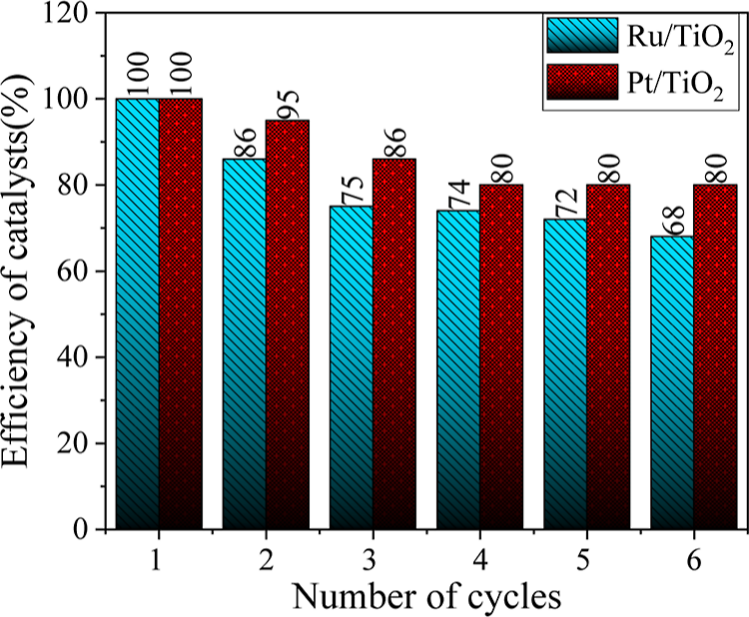
Efficiency of catalysts in their repeated use.

To study the durability of the Pt/TiO_2_ and Ru/TiO_2_ catalysts, cyclic tests were carried out. The used catalyst was thoroughly washed with distilled water to pH = 6–7 after each cycle test, separated from the solution, dried at 50 °C, and reused. After six cycles, the Pt/TiO_2_ and Ru/TiO_2_ catalysts retained 80 and 68% of their initial efficiency, respectively (Figure 8). The loss of catalytic activity compared to the initial cycle indicates that the catalysts can be reused well up to 5–6 times. At the same time, increasing the amount of catalyst after a drop in its efficiency will allow it to be used while maintaining the initial time of complete hydrolysis.

One of the important process parameters determining the time of complete hydrolysis is the amount of catalyst. The more catalysts, the higher the heating temperature of the NaBH_4_ solution, and consequently, the faster the process proceeds in proportion to the increase in the amount of catalyst. According to the research findings, increasing Ru and Pt loading boosts catalyst efficiency while decreasing activation energy.^34,38,39,45,64,65^ The question arises about the expediency of increasing the loading of noble metals in the catalyst. Such an increase is possible for cobalt and nickel, the cost of which is an order of magnitude lower. Generally, industrial catalysts based on noble metals have low loads, which reduce their cost. Since in real hydrogen generators the process is far from isothermal, it makes sense to use self-heating of the solution to accelerate hydrolysis. This will reduce both the amount of catalyst and the noble metal content. Hydrogen generation rates and activation energy from the NaBH_4_ hydrolysis with Ru- and Pt-based catalysts prepared in this work and catalysts described in the literature are compared in Table 1.

## 4 Conclusions

The hydrogen generation rate for the Ru/TiO_2_ catalyst decreases with increasing NaOH concentration, while for the Pt/TiO_2_ catalyst, it passes through the maximum. The maximum efficiency of the hydrolysis process in the presence of a Ru/TiO_2_ catalyst is achieved in an aqueous solution of NaBH_4_ and with a Pt/TiO_2_ catalyst in an aqueous-alkaline solution in the range of 0.5–2 M NaOH. This is confirmed by the obtained values of activation energies in aqueous and aqueous-alkaline solutions. It was shown that the nature of the effect of the influence of alkali on the rate of hydrolysis with Ru- and Pt-based catalysts is determined by the carrier material. The found activation energies do not fall out of the range of values given by other authors for catalysts with low Pt and Ru loading. The cost-effective Ru/TiO_2_ and Pt/TiO_2_ catalysts in this study, despite the low noble metal loading, demonstrate good efficiency for use in hydrogen production. The rate of hydrogen generation is determined by the requirements of its application and depends not only on the activity of the catalyst but also on the solution temperature, reactor mass, heat exchange conditions, and efficiency of mass transfer processes.

## Supporting Information Available

BET analysis results of pristine TiO_2_ powder; BET analysis results of Pt/TiO_2_ and Ru/TiO_2_ before and after first use in NaBH_4_ hydrolysis experiments; and comparison of activation energies and HGRs during hydrolysis of NaBH_4_ catalyzed by Pt and Ru catalysts (PDF)
