# Supplementary material for: Ruthenium and Platinum-Modified Titanium Dioxide Support for NaBH4 Hydrolysis
Source: ACS Omega. 2023 Sep 20;8(39):36100–8. doi: 10.1021/acsomega.3c04269 (PMC10552117; doi:10.1021/acsomega.3c04269)
Supplement: Supplementary file 1 — ao3c04269_si_001.pdf [file ao3c04269_si_001.pdf]

Supplementary Information

Ruthenium and Platinum Modified Titanium Dioxide Support for $NaBH_4$ Hydrolysis

Cigdem Tuc Altaf,[a] Valentina G. Minkina,[b]* Stanislav I. Shabunya,[b] Tuluhan O. Colak,[a] Nurdan Demirci Sankir, [a, c] * Mehmet Sankir,[a, c] * Vladimir I. Kalinin[b]

[a] Micro and Nanotechnology Graduate Program, TOBB University of Economics and Technology, Sogutozu Caddesi No 43 Sogutozu 06560 Ankara, Turkey

[b] A.V. Luikov Heat and Mass Transfer Institute of the National Academy of Sciences of Belarus, P. Brovka, 15. Minsk, 220072 Republic of Belarus

[c] Department of Materials Science and Nanotechnology Engineering, TOBB University of Economics and Technology, Sogutozu Caddesi No 43 Sogutozu 06560 Ankara, Turkey

Corresponding authors: *minkina@dnp.itmo.by * nsankir@etu.edu.tr * msankir@etu.edu.tr

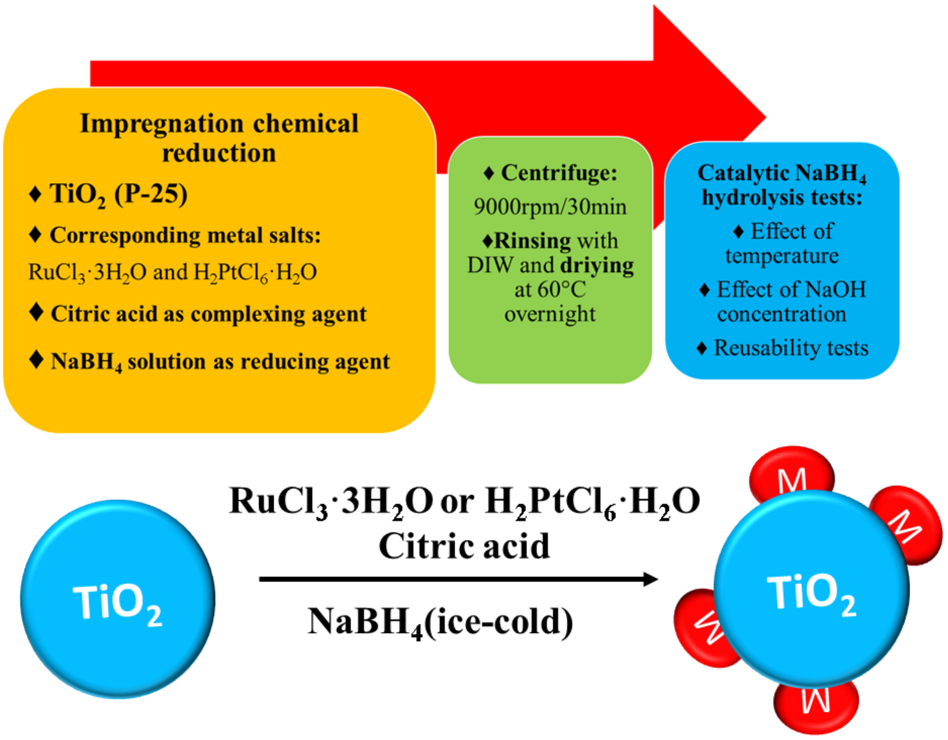

Figure S1. Flow chart for the experimental detail.

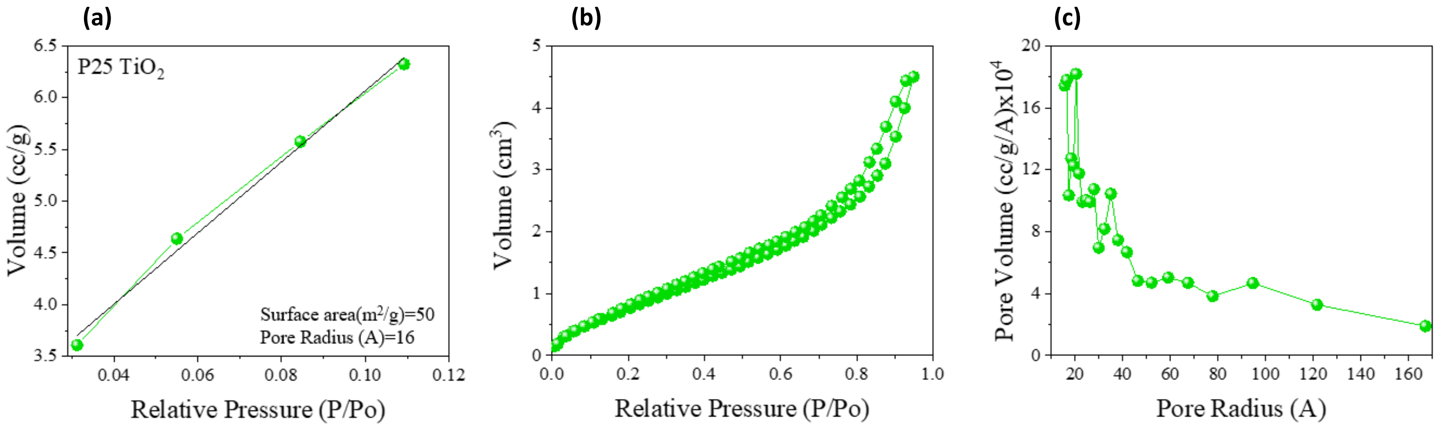

Figure S2. BET analysis results of pristine TiO_2 powder;(a) multi-point plots with respective fittings, (b) nitrogen adsorption/desorption isotherms, (c) average pore size distributions

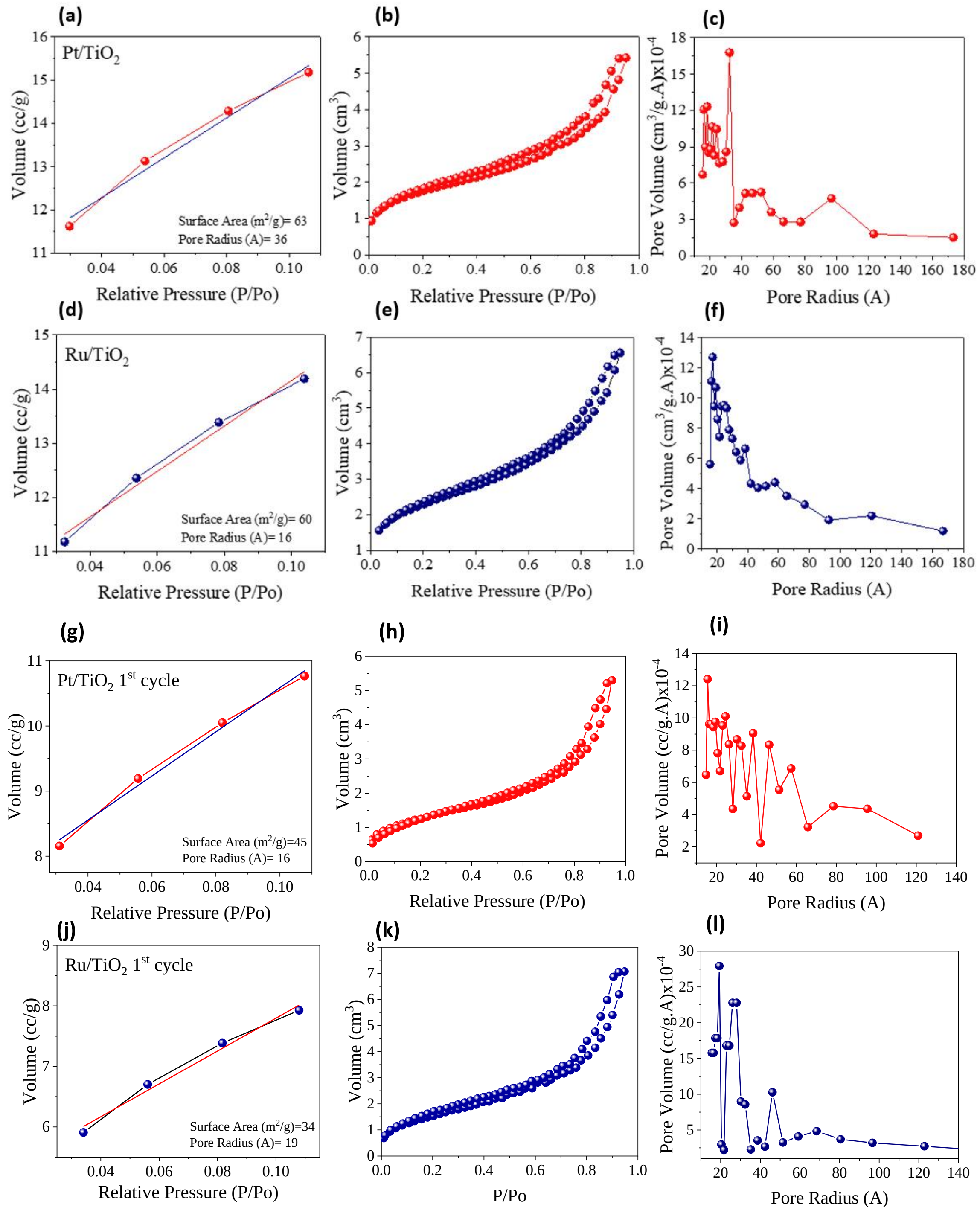

Figure S3. BET analysis results before use Pt/TiO_2 (a-c) and Ru/TiO_2 (d-f) in $NaBH_4$ hydrolysis experiments; (a) and (d) multi-point plots and their respective fittings, (b) and (e) nitrogen adsorption/desorption isotherms, (c) and (f) average pore size distributions. BET analysis results of Pt/TiO_2 and Ru/TiO_2 after 1st use in $NaBH_4$ hydrolysis experiments; (g) for

Pt/TiO2 and (j) for Ru/TiO2 multi-point plots and their respective fittings, (h) Pt/TiO2 and (k) Ru/TiO2 nitrogen adsorption/desorption isotherms, (i) Pt/TiO2 and (l) Ru/TiO2 average pore size distributions

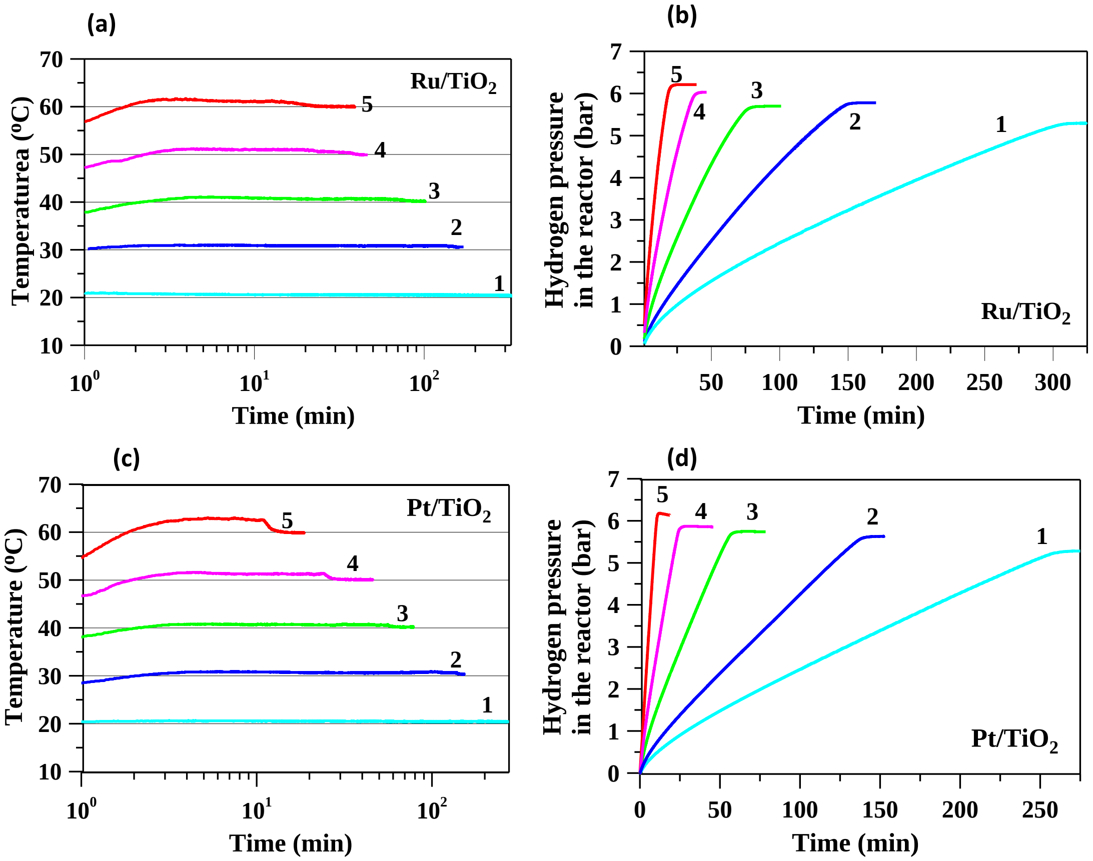

Figure S4. (a) Temperature and (b) Pressure history for Ru/TiO2, (c) Temperature and (d) Pressure history for Pt/TiO2 during the hydrolysis process *(1 – 20°C, 2 – 30°C, 3 – 40°C, 4 – 50°C, 5 – 60°C. 1.06 M NaBH4, 50 mg of the catalyst).*

Table S1. Comparison table of activation energies and HGRs during hydrolysis of $NaBH_4$ catalyzed by Pt and Ru catalysts

Catalyst	Me (Ru, Pt) (wt%)	$NaBH_4$ (wt%)	NaOH (wt%)	Cat. (mg)	E_a kJ.mol^{-1}	Average HGR at 25°C (mL min^{-1} g_{cat}^{-1})	Ref.
Pt/R-NH	2	0.5M	0.5M	41	41	110	[1]
Ru/Al_2O_3	5	12.5	1	41.8	41.8	70	[2]
Ru/TiO_2	0.145	5	2	55.9	55.9	39	[3]
Ru/$LiCoO_2$	1	10	5	20	68.5	3000	[4]
Pt/$LiCoO_2$	1	10	5	20	70.4	2700	[4]

References

[1] Feng X., Song Z., Guo T., Yang R., et al. Insights into the effect of surface functional groups on catalytic performance for hydrogen generation from sodium borohydride. RSC Advances. 2016. V. 6. 113260. doi.org/10.1039/C6RA25016E

[2] Su C.-C., Lu M.-C., Wang S.-L., Huang Y.-H. Ruthenium immobilized on Al2O3 pellets as a catalyst for hydrogen generation from hydrolysis and methanolysis of sodium borohydride. RSC Advances. 2012. V. 2. 2073–2079. doi.org/10.1039/C2RA01233B

[3] Wei L., Ma M., Wang D., Wang Q., et al. Hydrogen generation from the hydrolysis of sodium borohydride using TiO_2 supported Ru nanocatalysts prepared by photocatalytic reduction. Functional Materials Letters. 2018. V. 11 (4). 1850079 doi.org/10.1142/S1793604718500790

[4] Y. Wei, R. Wang, L. Meng, Y. Wang, et al. Hydrogen generation from alkaline $NaBH_4$ solution using a dandelion-like Co-Mo-B catalyst supported on carbon cloth. Int. J. of Hydrogen Energy. 2017. V. 42 (15). 9945–9951. doi.org/10.1016/j.ijhydene.2016.12.130
